# Association between endometrial thickness and cumulative live birth rate in one oocyte-retrieval cycle: a retrospective cohort study including 26,127 patients

**DOI:** 10.1186/s12884-026-08897-6

**Published:** 2026-03-05

**Authors:** Gege Ouyang, Huiying Xiao, Ning Li, Yue Niu, Yuan Fang, Jialin Zou, Dingying Zhao, Daimin Wei

**Affiliations:** 1 State Key Laboratory of Reproductive Medicine and Offspring Health, Center for Reproductive Medicine, Institute of Women, Children and Reproductive Health, Shandong University, Jinan, 250012 China; 2 Key Laboratory of Reproductive Endocrinology, Ministry of Education, Shandong University, Jinan, 250012 China; 3 National Research Center for Assisted Reproductive Technology and Reproductive Genetics, Jinan, 250012 Shandong China

**Keywords:** Endometrial thickness, Cumulative live birth rate, Live birth rate, Embryo transfer stage, Fresh embryo transfer

## Abstract

**Background:**

This retrospective study aimed to evaluate the association between endometrial thickness (EMT) on the day of human chorionic gonadotropin (hCG) trigger and the cumulative live birth rate (CLBR) across one oocyte-retrieval cycle.

**Methods:**

This single-center retrospective cohort study included 26,127 women undergoing their first cycles of in vitro fertilization with or without intracytoplasmic sperm injection with autologous oocytes between January 2013 and December 2019. EMT on the hCG trigger day was analyzed both categorically (Group 1: <8.0, Group 2: 8.0–10.9, Group 3: 11.0–13.9, Group 4: ≥14.0 mm) and as a continuous variable in 1–2 mm increments. The primary outcome was the CLBR per retrieval cycle. Subgroup analyses were stratified by embryo number and stage at fresh transfer, maternal age, and the number of oocytes retrieved. Multivariable logistic regression analyses assessed the association between EMT and pregnancy outcomes.

**Results:**

The CLBR increased with each millimeter of EMT, reaching a maximum at 15 mm before slightly declining. Across EMT categories, the CLBR rose from 45.4% (< 8.0 mm) to 74.6% (≥ 14.0 mm). Live birth rates following fresh embryo transfer were 27.0%, 44.0%, 53.8%, and 58.0%for groups 1 to 4, respectively. Conversely, the absolute gains from subsequent frozen embryo transfers remained relatively constant, at 18.4%, 19.5%, 17.6%, and 16.6%. A positive association between EMT and CLBR was evident in all subgroups. In fresh cycles, thicker EMT was associated with lower rates of biochemical miscarriage and ectopic pregnancy (*P* < .001). And a significant interaction was observed between EMT and embryo transfer strategy on live birth rate (*P* < .001).

**Conclusions:**

EMT on the day of hCG trigger is positively associated with CLBR within one oocyte- retrieval cycle, with optimal outcomes around 15 mm. This association is predominantly driven by fresh-transfer outcomes, whereas the absolute contribution from subsequent frozen transfers is relatively stable.

**Supplementary Information:**

The online version contains supplementary material available at 10.1186/s12884-026-08897-6.

## Introduction

In patients undergoing in vitro fertilization (IVF) and intracytoplasmic sperm injection (ICSI), the measurement of endometrial thickness (EMT) by ultrasound is routinely conducted prior to embryo transfer. EMT is the most widely recognized surrogate marker of endometrial receptivity and a significant predictor of pregnancy outcomes in assisted reproductive technology (ART) [1, 2].

A number of observational studies have investigated the association between EMT and the likelihood of achieving pregnancy or live birth after IVF, with conflicting results. A thin endometrial lining, commonly defined as < 6 mm, < 7 mm, or < 8 mm, has been associated with lower implantation and pregnancy rates, as well as an increased risk of spontaneous abortion [3–8]. Conversely, EMT exceeding 7–8 mm has generally been associated with improved IVF outcomes [9–14]. Several studies have even concluded that EMT have limited predictive value for IVF outcomes [15–18].

Most of these studies, however, focused on the outcomes of a single cycle of embryo transfer. Given that the majority of patients need multiple transfers, the cumulative live birth rate (CLBR) is increasingly regarded as a more comprehensive patient-centered endpoint that reflects IVF success [19–22]. Despite its growing importance, few investigations have systematically examined the association between EMT and CLBR within one oocyte- retrieval cycle while accounting for potential effect modifiers.

In this cohort study, we examined the association between EMT on the day of human chorionic gonadotropin (hCG) trigger and the CLBR within a single oocyte-retrieval cycle. We further decomposed CLBR into outcomes from the fresh transfer and the subsequent frozen transfers to elucidate how between-group differences in fresh-cycle EMT translate into cumulative differences. And we also assessed whether this association differed by embryo number and stage at the fresh transfer, maternal age and oocyte yield.

## Materials and methods

### Study design and populations

This retrospective cohort study was conducted at the Center of Reproductive Medicine of Shandong University. Women aged 20–40 years who underwent their first IVF attempt with or without ICSI using autologous oocytes between January 2013 and December 2019 were included. The study protocol was approved by the Institutional Ethics Committee of the Center for Reproductive Medicine of Shandong University.

The exclusion criteria were women who underwent preimplantation genetic testing (PGT) cycles, women with a history of recurrent pregnancy loss, defined as 2 or more previous pregnancy losses, uterine abnormalities such as submucosal myoma, adenomyosis, or intrauterine adhesion, and those with loss to follow-up or missing core data.

A complete cycle was defined as all fresh and subsequent cryopreserved embryo transfer attempts associated with a single oocyte retrieval episode. Baseline characteristics, including patients’ demographics, were extracted from the clinical electronic medical database. The minimum follow-up time from the start of ovarian stimulation was 1 year.

### EMT assessment

The EMT was measured using transvaginal ultrasound with the greatest distance in the midsagittal plane between the anterior and posterior endometrial-myometrial interfaces. To ensure accuracy and minimize measurement errors, two highly trained clinicians were involved in the process: one acted as the operator, while the other served as the checker and recorder. EMT measurement was taken on the day of hCG trigger during ovarian stimulation, which represented the endometrial response under controlled ovarian stimulation at the time when a fresh transfer decision is typically made. To enhance the clinical applicability of our study, we categorized EMT into four groups based on the 5th (8 mm), 50th (11 mm), and 95th (14 mm) percentiles of the study cohort (< 8, 8-10.9, 11-13.9, and ≥ 14 mm).

### Embryo culture, evaluation, and selection for transfer

The oocytes were inseminated approximately 4 to 6 h after follicular aspiration using either a conventional method or intracytoplasmic sperm injection, depending on sperm quality. Blastocyst culture was performed with sequential media. On day 3, embryos were transferred from cleavage media to blastocyst media. A good quality cleavage stage embryo was defined as embryos with 7–10 cells and a morphological score of 4 or 3, according to the Puissant criteria [23]. A good quality blastocyst was defined as a blastocyst with expansion stage 4 or higher and an inner cell mass score of B or better according to Gardner criteria [24]. Up to two good quality embryos were selected for fresh transfer, and any remaining embryos were cryopreserved by vitrification. For statistical analysis, cleavage stage and blastocyst transfers were included, with day-5 and day-6 blastocysts transfers analyzed together.

### Follow-up and outcomes measures

The primary outcome was cumulative live birth, which was defined as achieving one live birth following one oocyte-collection cycle. Prespecified secondary outcomes included live birth, biochemical pregnancy, clinical pregnancy, pregnancy loss and ectopic pregnancy after fresh embryo transfer. Live birth was defined as delivery of any viable infant at 28 weeks or more of gestation. Biochemical pregnancy was defined as pregnancy confirmed by serum hCG levels ≥ 10 mIU/ml. Clinical pregnancy was defined as the presence of a gestational sac by ultrasound scan at 7–8 weeks gestation, which includes intrauterine, ectopic, and heterotopic pregnancies. Pregnancy loss was calculated as the number of conceptions (positive hCG tests) minus the number of live births, divided by the total number of conceptions.

### Statistical analysis

We first performed the descriptive statistics for baseline factors that may influence pregnancy outcomes. Continuous variables were expressed as means±standard deviation for normally distributed continuous variables, and median with interquartile range for nonnormally distributed continuous variables. Comparisons among different groups were performed using ANOVA, Welch’s ANOVA or the Kruskal–Wallis test, depending on data distribution. Categorical variables were summarized as frequencies (percentages) and analyzed using the Pearson chi-square test.

For CLBR analyses, subsequent frozen-thawed transfers were restricted to blastocysts to standardize embryo stage across frozen embryo transfer cycles, consistent with clinical practice. women who had remaining available embryos were counted as no live birth in subsequent embryo transfers. Subgroup analyses were performed by the number and development stage of the transferred embryos (one cleavage stage embryo, two cleavage stage embryos, and one blastocyst), maternal age, and number of oocytes retrieved to assess potential effect modification on these associations.

Finally, Multivariable logistic regression analysis was conducted to assess the association between EMT and pregnancy outcomes, with individuals having an EMT of 8–10.9 mm as the reference group. Potential confounders identified from prior literature and clinical expertise included in the model were maternal age, body mass index, infertility type, IVF indication, basal follicle stimulating hormone, basal luteinizing hormone, basal estradiol, ovarian stimulation protocol, days of ovarian stimulation, total gonadotropin dose, number of oocytes retrieved, fertilization method, number of embryos transferred, stage of embryos transferred, and the interaction between the embryo transfer policy and EMT. The results were presented with an adjusted odds ratio (OR) and 95% confidence intervals (CI). All analyses were performed using SPSS, (Ver. 26.0, IBM, Chicago, IL, USA). A two-sided *P* value < 0.05 was considered statistically significant.

## Results

### Baseline characteristics

A total of 26,127 cycles were included in the analysis, which were categorized into four groups based on EMT on the day of hCG trigger: Group 1 (< 8 mm, *n* = 831), Group 2 (8–10.9 mm, *n* = 10,075), Group 3 (11–13.9 mm, *n* = 13,373), and Group 4 (≥ 14 mm, *n* = 1,848). Baseline demographic and treatment characteristics across the EMT groups are presented in Table 1. The EMT in the study ranged from 4 to 21 mm.

**Table 1:** Patient baseline characteristics and cycle parameters by endometrial thickness

	<8 mm (*n* = 831)	8–10.9 mm (*n* = 10075)	11–13.9 mm (*n* = 13373)	≥ 14 mm (*n* = 1848)	*P* value
Age (years)	32.88 ± 4.48	31.68 ± 4.33	30.83 ± 4.24	30.85 ± 4.30	<0.001^b^
BMI(kg/m²)	23.52 ± 3.36	23.41 ± 3.53	23.40 ± 3.51	23.71 ± 3.61	0.004^a^
Infertility type, No. (%)					<0.001^d^
Primary infertility	245(29.2)	4456(44.2)	7338(54.9)	1012(54.7)	
Secondary infertility	593(70.8)	5623(55.8)	6036(45.1)	839(45.3)	
IVF Indication, No. (%)					<0.001^d^
Tubal factor	600 (72.2)	7037 (69.8)	8844 (66.1)	1193 (64.6)	
Male factor	49 (5.9)	861 (8.5)	1510 (11.3)	205 (11.1)	
Multiple factors	156 (18.8)	1828(18.1)	2638 (19.7)	404 (21.9)	
Unexplained factors	16 (1.9)	234(2.3)	270 (2.0)	36 (1.9)	
Other	10(1.2)	115(1.1)	111(0.8)	10(0.5)	
Basal FSH (IU/L)	7.49 ± 3.00	7.06 ± 2.26	6.99 ± 2.11	7.00 ± 1.93	<0.001^b^
Basal LH (IU/L)	4.43(3.27,5.87)	4.50(3.40,5.85)	4.56(3.44,5.95)	4.53(3.44,5.88)	0.051^c^
Basal E2(IU/L)	38.46 ± 17.48	37.04 ± 15.97	35.25 ± 14.95	34.92 ± 14.75	<0.001^b^
Ovarian stimulation protocol, No. (%)					<0.001^d^
GnRH agonist long	326 (39.2)	5193 (51.5)	8507 (63.6)	1340 (72.5)	
GnRH agonist short	329 (39.6)	3335 (33.1)	3039 (22.7)	314(17.0)	
GnRH antagonist	112 (13.5)	1354 (13.4)	1713 (12.8)	176 (9.5)	
Other	64 (7.7)	193 (1.9)	114 (0.9)	18 (1.0)	
Days of ovarian stimulation	9.79 ± 2.41	10.16 ± 2.17	10.69 ± 2.15	11.02 ± 2.17	<0.001^b^
Total gonadotropin dose (IU)	1980.07 ± 980.41	1978.01 ± 918.13	2038.13 ± 932.90	2157.46 ± 994.58	<0.001^b^
EMT on hCG trigger day (mm)	6.99 ± 0.65	9.41 ± 0.72	11.96 ± 0.80	14.79 ± 1.14	<0.001^b^
No. of oocytes retrieved	7.0 (4.0,11.0)	9.0 (6.0,12.0)	10.0 (7.0,13.0)	10.0 (7.0,13.0)	<0.001^c^
No. of D3 good quality embryos	2.0 (1.0,4.0)	3.0 (2.0,5.0)	3.0 (2.0,5.0)	3.0 (2.0,5.0)	<0.001^c^
Fertilization method, No. (%)					<0.001^d^
IVF	676 (81.3)	7582(75.3)	9475 (70.9)	1295 (70.1)	
ICSI	133 (16.0)	2086 (20.7)	3434 (25.7)	491 (26.6)	
IVF/IVSI	22 (2.6)	407(4.0)	464 (3.5)	62(3.4)	
No. and stage of embryo transferred, No. (%)					<0.001^d^
One cleavage-stage embryo	82 (9.9)	717 (7.1)	837 (6.3)	101 (5.5)	
Two cleavage-stage embryos	548 (65.9)	7450 (73.9)	10,371 (77.6)	1417 (76.7)	
One blastocyst	198 (23.8)	1882 (18.7)	2134 (16.0)	321 (17.4)	
Two blastocysts	3 (0.4)	26 (0.3)	31 (0.2)	9 (0.5)	
No. of good quality embryos transferred, No. (%)					<0.001^d^
0	413 (49.7)	5862(58.2)	8805 (65.8)	1170 (63.3)	
1	168 (20.2)	1469 (14.6)	1438 (10.8)	196 (10.6)	
2	250 (30.1)	2744 (27.2)	3130 (23.4)	481 (26.0)	
No. of embryos cryopreserved	1.0(0.0–2.0)	1.0(0.0–3.0)	2.0(0.0–3.0)	1.0(0.0–3.0)	<0.001^c^

Data are presented as mean ± standard deviation, median (interquartile range) and *n* (%). *BMI*  body mass index, *FSH*  follicle stimulating hormone, *LH*  luteinizing hormone, *E2* estradiol, *GnRH*  gonadotropin releasing hormone, *EMT* endometrial thickness, *hCG*  human chorionic gonadotropin, *IVF*  in vitro fertilization, *ICSI*  intracytoplasmic sperm injection

a One-way ANOVA

b Welch’s ANOVA

c Kruskal-Wallis test

d Pearson chi-square test

Among the 26,127 transfer cycles, the mean patient age was 31.2 ± 4.31 years and the mean maternal body mass index (BMI) was 23.4 ± 3.52 kg/m². Patients with a thick endometrial lining (Group 3 or 4) were younger than those with a thin endometrium, had a higher proportion of primary infertility, and were more likely to present with non-tubal infertility factors (e.g., male infertility). These patients also underwent longer ovarian stimulation, received higher gonadotropin doses, and yielded more oocytes in fresh cycles. However, their proportion of good quality fresh embryos transferred was relatively lower. Double cleavage-stage embryo transfer was the predominant procedure across all cycles.

In an exploratory analysis among patients who underwent at least one subsequent FET, trigger-day EMT showed a moderate positive correlation with EMT measured during FET endometrial preparation (*r* = .6341, *P* < .0001, Supplemental Fig. 1). In addition, maternal age, BMI, basal follicle stimulating hormone levels, basal estradiol levels, infertility types, IVF indications, ovarian stimulation protocols, days of ovarian stimulation, total gonadotropin dose, fertilization methods, the stage and number of embryos transferred were significantly different among groups.

### Primary outcome

**Fig. 1:**
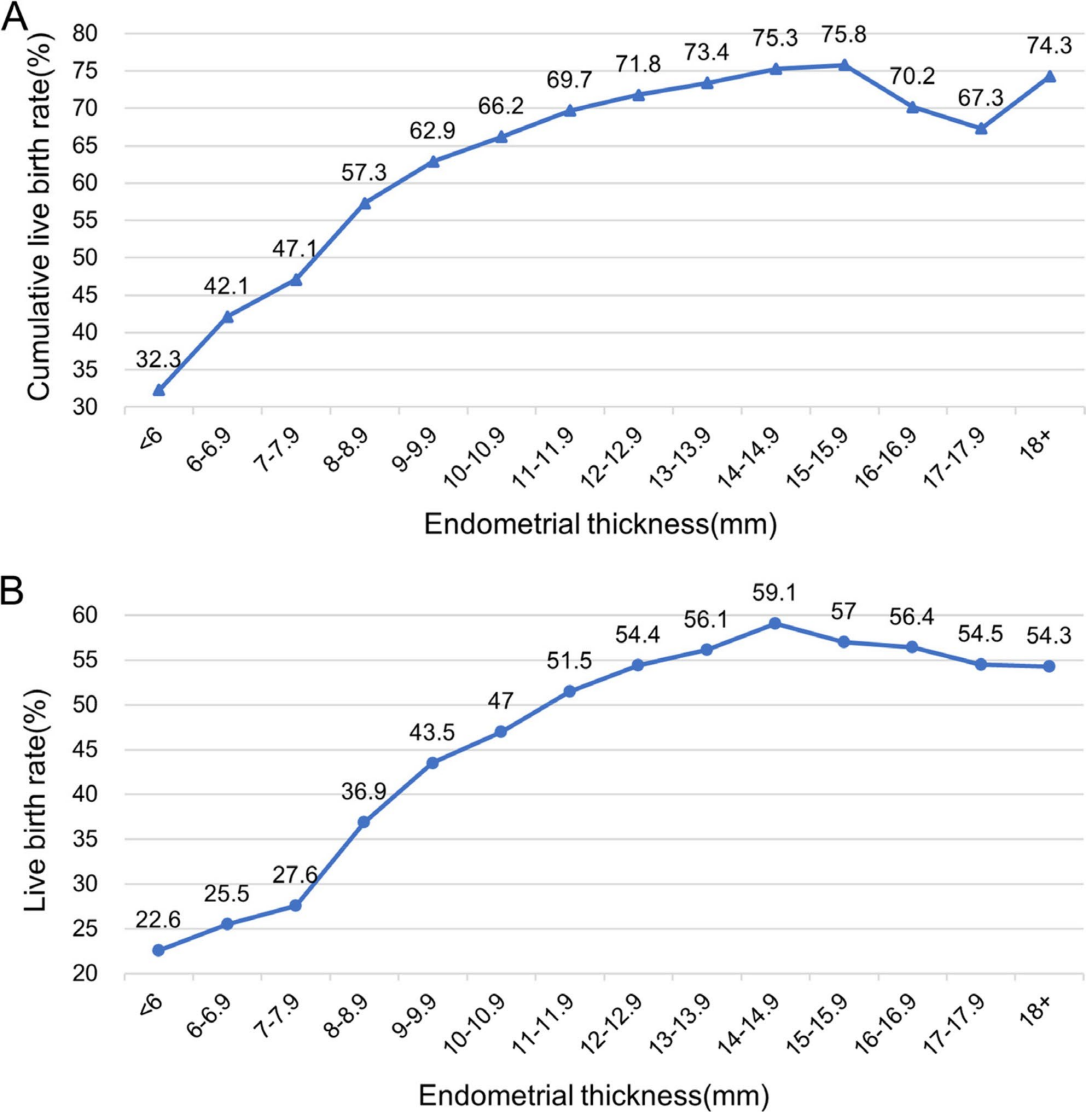
Association between endometrial thickness on the hCG trigger day and (**A**) cumulative live birth rate (CLBR) and (**B**) live birth rate (LBR) after fresh embryo transfer, in 1-mm increments

**Fig. 2:**
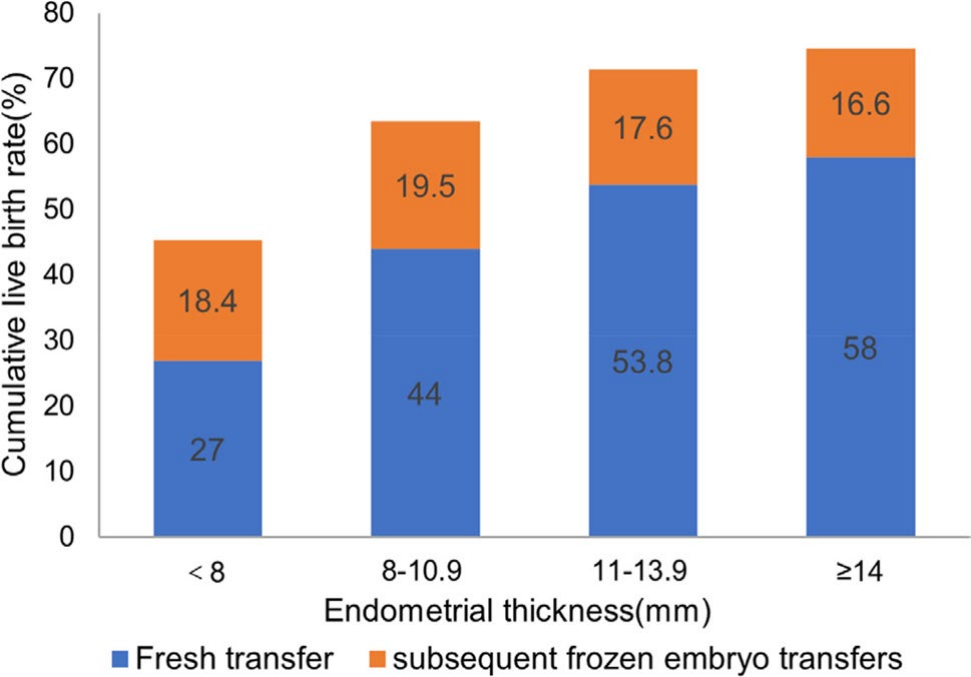
Decomposition of the CLBR into outcomes from the fresh transfer and subsequent frozen embryo transfers

With each 1-mm increase in EMT, the CLBR improved progressively, peaked at 75.8% with an EMT of 15 mm, and then declined slightly (Fig. 1A). The absolute number of cycles and pregnancy outcomes are shown in Supplementary Table 1. In the categorical analysis, the CLBR increased across EMT groups, rising from 45.4% in Group 1 to 63.5%, 71.4%, and 74.6% in Groups 2–4 (*P* < .001, Table 2). CLBR decomposition showed that between-group differences were driven mainly by fresh-cycle performance (Fig. 2). Fresh-cycle LBR were 27.0%, 44.0%, 53.8%, and 58.0% for groups 1 to 4, respectively, whereas the absolute gain from subsequent FETs remained stable across groups, at 18.4%, 19.5%, 17.6%, and 16.6% across Groups 1–4.

**Table 2:** CLBR and clinical outcomes after fresh embryo transfer by endometrial thickness

	<8 mm (*n* = 831)	8–10.9 mm (*n* = 10075)	11–13.9 mm (*n* = 13373)	≥ 14 mm (*n* = 1848)	*P* value*
Primary outcome					
Cumulative Live birth†	377(45.4)	6392(63.5)	9545(71.4)	1379(74.6)	<0.001
Secondary outcomes					
Live birth rate	224(27.0)	4430(44.0)	7191(53.8)	1072(58.0)	<0.001
Biochemical pregnancy	384(46.2)	6142(61.0)	9227(69.0)	1319(71.4)	<0.001
Clinical pregnancy	306(36.8)	5372(53.3)	8302(62.1)	1216(65.8)	<0.001
Pregnancy loss	142(17.1)	1539(15.3)	1907(14.3)	234(12.7)	0.002
Biochemical miscarriage	78(9.4)	770(7.6)	925(6.9)	103(5.6)	<0.001
Clinical pregnancy loss	64(7.7)	769(7.6)	982(7.3)	131(7.1)	0.765
First trimester pregnancy loss	55(6.6)	684(6.8)	884(6.6)	112(6.1)	0.717
Second trimester pregnancy loss	9(1.1)	85(0.8)	98(0.7)	19(1.0)	0.330
Ectopic pregnancy	18(2.2)	173(1.7)	129(1.0)	13(0.7)	<0.001

Data are presented as *n* (%)

†1,777 patients who had remaining available cryopreserved embryos were treated as no live birth in subsequent embryo transfers; 16 patients who underwent frozen transfer with cleavage-stage embryos were excluded

*P values for differences in pregnancy outcome rates across endometrial thickness strata. All comparisons were analyzed using Fisher’s exact test. Statistical significance after Bonferroni correction was determined by a *P*-value of < 0.0167 (0.05/3)

**Fig. 3:**
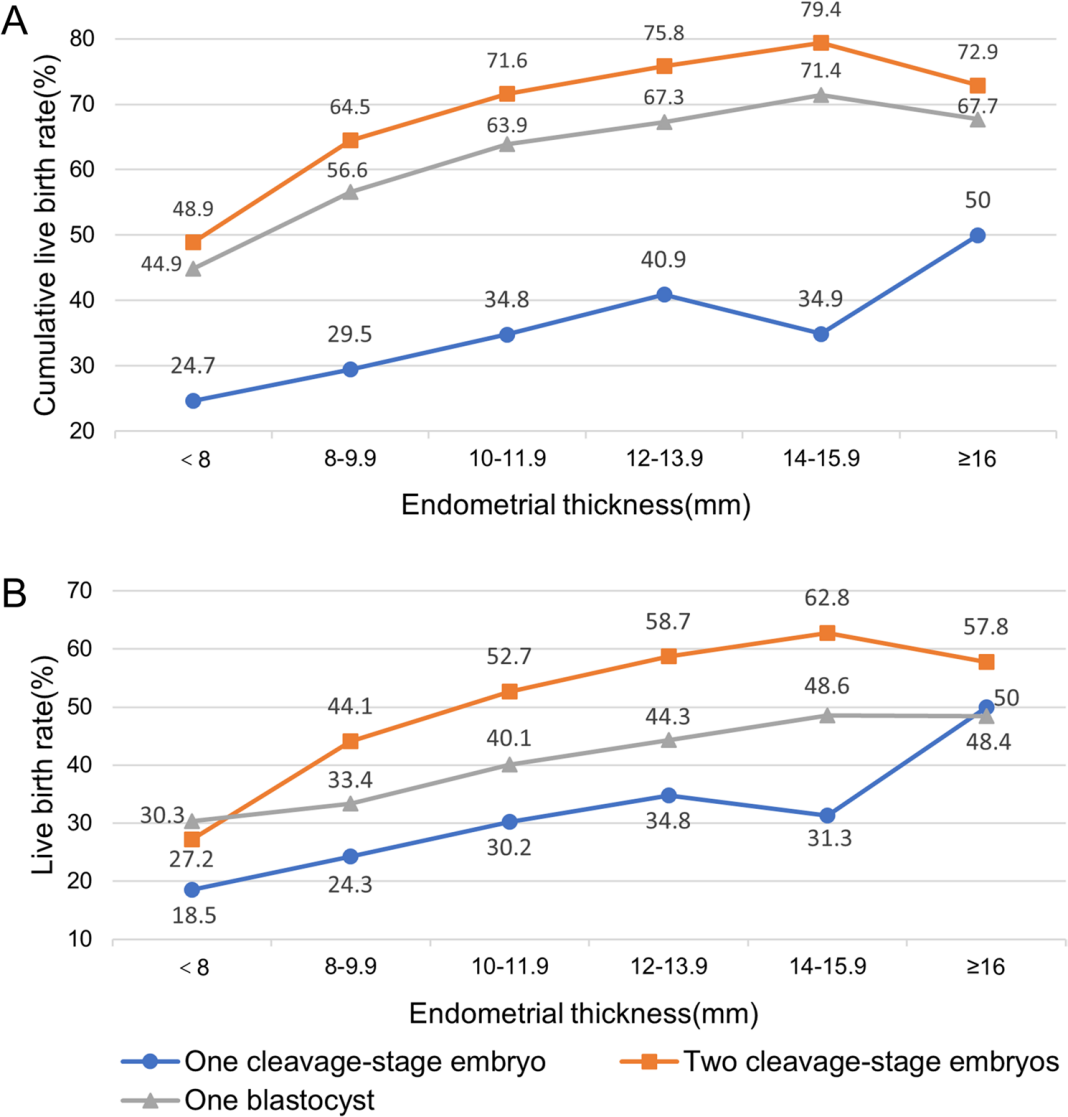
Association between endometrial thickness on the hCG trigger day and (**A**) CLBR or (**B**) LBR after fresh embryo transfer according to embryo trasnfer policy, in 2-mm increments. Note: The sample size of two blastocyst transfer (*n*=69) is not powered to detect differences in clinical outcomes; Stimates for the ≥16 mm category in the one cleavage-stage group (*n*=18) should be interpreted with caution due to the small sample size

We additionally stratified the EMT–CLBR curves by both embryo stage and embryo number (Fig. 3A). A positive association between EMT and CLBR was observed across all three subgroups.

The relationship between EMT and CLBR across different age groups (< 30, 30–34 and 35+) was illustrated in Supplemental Fig. 1. In each age group, CLBR increased significantly with the increase in EMT. When EMT exceeded 16 mm, CLBR levels converged across age groups.

Since both EMT and CLBR were associated with the number of oocytes retrieved, we further analyzed CLBR by the number of oocytes retrieved (< 4, 4–8,9–12, and ≥ 13; Supplemental Fig. 2) and observed significant improvements in CLBR with increasing EMT across all subgroups.

### Secondary outcomes

The association between trigger-day EMT and fresh-cycle LBR mirrored that for CLBR. LBR increased with EMT, peaked at ~ 15 mm (59.1%), and then slightly declined (Fig. 1B). In stratified analyses by embryo stage and number transferred (Fig. 3B), LBR generally increased with EMT across all strata. Overall, single-blastocyst transfer achieved higher LBR than single cleavage-stage transfer across most EMT categories. Notably, in thin endometrium (< 8 mm), single-blastocyst transfer yielded higher LBR than double cleavage-stage transfer, whereas for EMT ≥ 8 mm, double cleavage-stage transfer showed higher LBR. A significant interaction was detected between EMT and transfer strategy (*P* < .001, Table 3).

**Table 3:** Interaction between embryo stage and endometrial thickness on live birth rate after fresh embryo transfer

	adjusted OR (95%CI): two cleavage embryos vs. one blastocyst	*P* value	*P* value for interaction
Live birth fresh	1.24(0.86–1.77)	0.253	<0.001
EMT<8 mm	1.16(0.80–1.69)	0.436	
EMT≥8 mm	0.62(0.58–0.67)	<0.001	

The covariables in the overall regression models were maternal age, body mass index, infertility type, IVF indication, basal follicle stimulating hormone, basal luteinizing hormone, basal estradiol, ovarian stimulation protocol, days of ovarian stimulation, total gonadotropin dose, number of oocytes retrieved, fertilization method, number of embryos transferred, stage of embryos transferred, endometrial thickness (<8 mm vs. ≥8 mm), and the interaction between the embryo transfer policy and EMT

For each endometrial thickness subgroup, the regression model included covariables of maternal age, body mass index, infertility type, IVF indication, basal follicle stimulating hormone, basal luteinizing hormone, basal estradiol, ovarian stimulation protocol, days of ovarian stimulation, total gonadotropin dose, number of oocytes retrieved, fertilization method, number of embryos transferred, stage of embryos transferred

In fresh transfer cycles, a thicker endometrium was associated with higher rates of live birth (27.0%, 44.0%, 53.8%, and 58.0% for Groups 1–4, respectively; *P* < .001), biochemical pregnancy and clinical pregnancy, as well as lower pregnancy loss rates. Group 1 also had a significantly higher prevalence of biochemical miscarriage (9.4%, 7.6%, 6.9%, and 5.6% for Groups 1–4, respectively; *P* < .001) and ectopic pregnancy (2.2%, 1.7%, 1.0%, and 0.7% for Groups 1–4, respectively; *P* < .001) compared to the other groups. No significant differences were observed among the groups for clinical pregnancy loss, first trimester pregnancy loss, or second trimester pregnancy loss (Table 2).

### Results of multivariable logistic regression analysis

The results of logistic regression analyses are presented in Table 4. After adjusting for potential confounders, EMT remained significantly associated with CLBR. Group 1 (EMT < 8 mm) had a significantly lower CLBR (adjusted OR = 0.56, 95% CI: 0.48–0.65), compared with the reference group (Group 2: 8–10.9 mm). CLBR increased progressively with EMT, with Group 3 (11–13.9 mm) showing an adjusted OR of 1.28 (95% CI: 1.20–1.36) and the highest CLBR (adjusted OR = 1.52, 95% CI: 1.35–1.71) for Group (≥ 14 mm). In addition to CLBR, EMT was also independently predictive of other clinical outcomes, including live birth, biochemical pregnancy, clinical pregnancy, pregnancy loss and ectopic pregnancy.

**Table 4:** Multivariable regression analysis by endometrial thickness

	Group1(<8) vs. Group2(8-10.9)		Group3(11-13.9) vs. Group2(8-10.9)		Group4(≥ 14) vs. Group2(8-10.9)	
	OR (95% CI)	*P* value	OR (95% CI)	*P* value	OR (95% CI)	*P* value
Primary outcome						
Cumulative Live birth	0.56(0.48–0.65)	<0.001	1.28(1.20–1.36)	<0.001	1.52(1.35–1.71)	<0.001
Secondary outcomes						
Live birth rate	0.53(0.45–0.62)	<0.001	1.35(1.28–1.43)	<0.001	1.60(1.44–1.77)	<0.001
Biochemical pregnancy	0.62(0.53–0.71)	<0.001	1.30(1.23–1.37)	<0.001	1.42(1.27–1.59)	<0.001
Clinical pregnancy	0.57(0.49–0.66)	<0.001	1.31(1.24–1.38)	<0.001	1.51(1.36–1.68)	<0.001
Pregnancy loss	1.22(0.95–1.56)	0.115	0.91(0.83–1.01)	0.080	0.72(0.58–0.89)	0.002
Biochemical miscarriage	0.98(0.75–1.28)	0.862	0.97(0.88–1.08)	0.601	0.91(0.75–1.10)	0.314
Clinical pregnancy loss	0.94(0.71–1.26)	0.683	0.98(0.88–1.09)	0.728	0.86(0.70–1.06)	0.166
First trimester pregnancy loss	1.24(0.62–2.48)	0.553	0.92(0.68–1.23)	0.564	1.26(0.76–2.09)	0.376
Second trimester pregnancy loss	1.11(0.92–1.34)	0.291	0.94(0.87–1.01)	0.094	0.80(0.69–0.93)	0.003
Ectopic pregnancy	1.28(0.78–2.11)	0.325	0.57(0.45–0.73)	<0.001	0.43(0.25–0.77)	0.004

*OR*  odds ratio, *CI*  confidence interval

Statistical significance after Bonferroni correction was determined by a *P*-value of < 0.0167 (0.05/3)

## Discussion

In this large retrospective cohort of 26,127 first IVF/ICSI cycles, EMT on the day of hCG trigger was positively associated with the CLBR within a single oocyte-retrieval cycle, with CLBR peaking at around 15 mm. The relationship was driven predominantly by outcomes in fresh cycles, whereas the absolute contribution from subsequent frozen embryo transfers was relatively stable across EMT strata.

These findings are consistent with a large dataset reporting a non-linear relationship between EMT on hCG trigger day and CLBR [25]. Given that FET occur in a different hormonal milieu from fresh cycles, we explored whether EMT phenotype might persist across cycles and observed a moderate correlation between fresh-cycle EMT and EMT recorded in FET cycles. However, heterogeneity in FET timing and preparation protocols limits inference. Accordingly, when CLBR is used as a patient-centered endpoint, trigger-day EMT should be interpreted as a marker of fresh-cycle endometrial development under supraphysiologic stimulation, rather than endometrial status in subsequent FET cycles.

Decomposition of the CLBR revealed that suboptimal EMT (< 8 mm) primarily limits cumulative outcomes through its impact on fresh transfer performance. One plausible explanation is that the supraphysiologic hormonal environment during ovarian stimulation may be more prone to impaired receptivity or endometrial–embryo asynchrony in thinner linings, whereas programmed or natural endometrial preparation in FET may partially normalize the hormonal environment and reduce the carryover from the fresh cycle. Clinically, these findings suggest that patients with thin EMT in the fresh cycle may benefit from strategies aimed at optimizing endometrial receptivity to improve cumulative outcomes, such as promoting endometrial thickening, or considering a freeze-all approach. However, these hypotheses require further prospective validation before practice recommendations can be made.

Stratified analyses accounting for embryo stage and number transferred suggested the EMT–CLBR association remained consistent within each stratum. Notably, Single-blastocyst transfer showed higher CLBR than single cleavage-stage transfer when combined with supernumerary single blastocyst vitrification, consistent with one recent RCT reporting modest advantages for single-blastocyst transfer in good-prognosis patients [26]. Our findings also suggested that two cleavage-stage embryos transfer resulted in higher CLBR than single-blastocyst transfer across EMT categories. Although single blastocyst transfer generally yields higher pregnancy rates per transfer compared to single cleavage-stage embryos [27–29], such benefits may not fully offset the reduced number of embryos transferred compared to DET. Importantly, these findings should not be interpreted as advocating double-embryo transfer, as contemporary guidelines prioritize single-embryo transfer to minimize risks of multiple gestations [29, 30]. Beyond transfer policy, maternal age and oocyte yield likely influence the association between EMT and outcomes by affecting embryo quality and selection [31–33]. Nevertheless, the relationship between EMT and outcome remained robust across these subgroups, demonstrating that the endometrial environment plays a significant role independent of embryo quantity or selection.

In addition, we observed a significant interaction between trigger-day EMT and embryo-transfer policy on fresh-cycle LBR. We hypothesize that extended blastocyst culture may enrich for embryos with higher implantation potential [34, 35], which could partially attenuate the adverse association between thin EMT (< 8 mm) and fresh-transfer success. This finding, however, should be interpreted cautiously due to the limited sample size in the extreme EMT strata, which make estimates less stable. Larger prospective studies are necessary to determine whether blastocyst transfer truly improves fresh-cycle LBR in women with thin EMT.

Regarding LBR after fresh embryo transfer, it increased with EMT, peaking around 15 mm, and declining slightly in our data. Interestingly, studies have reported divergent upper EMT thresholds for optimal LBR. Mahutte et al. observed that LBR plateaued between 10 and 12 mm [12], whereas Schmiech et al. reported improvements only up to 9 mm [10]. Such discrepancies across studies may reflect heterogeneity in study design, patient characteristics, measurement timing, embryo stage, stimulation protocols, or statistical modeling approaches. Our observation that an excessively thick endometrium may negatively affect IVF outcomes aligns with previous reports [10, 36], and the underlying biological mechanisms remain unclear. Whether LBR truly plateaus or declines beyond an upper threshold remains uncertain and warrants confirmation.

Increasing trigger-day EMT was also associated with higher biochemical and clinical pregnancy, together with lower overall pregnancy loss in fresh-transfer cycles. Notably, we observed biochemical miscarriage was significantly higher with thin EMT, while clinical miscarriage rates did not differ across EMT strata. This result is consistent with Gallos et al. which also reported that thin endometrium was associated with increased biochemical pregnancy loss [9]. This supports the interpretation that suboptimal EMT mainly compromises implantation establishment and early placentation stability rather than later gestation. We also observed a higher ectopic pregnancy rate in the thinnest EMT group, consistent with prior studies showing an inverse association between EMT and ectopic pregnancy risk after fresh embryo transfer [37]. However, the underlying mechanisms require further investigation.

The strengths of this study include its large sample size, a comprehensive evaluation of both cumulative and fresh cycle outcomes, and decomposition of fresh versus frozen contributions, which together enhance the reliability and clinical relevance of the findings. However, several limitations should be noted. First, the observational retrospective design precludes causal inference between EMT and CLBR due to inherent confounding and bias. Although we adjusted for various potential confounders, unmeasured or unknown factors, such as embryo quality grading, endometrial pattern or sub-endometrial Doppler flow [38], may still influence the results. Second, our findings are most applicable to oocyte-retrieval cycles that include fresh embryo transfer, and may limit generalizability to modern settings using PGT or freeze-all policies where embryo transfer occurs in a different hormonal environment. Furthermore, our analysis was limited to cycles that proceeded to embryo transfer. This may underestimate the effect of thin endometrium on IVF outcomes, as some cycles with suboptimal EMT may have been cancelled. Finally, treating patients with remaining cryopreserved embryos as having no live birth may introduce informative censoring and downwardly bias CLBR in certain strata.

## Conclusion

EMT on the day of hCG trigger was positively associated with CLBR within one oocyte-retrieval cycle, with an observed peak around 15 mm. The gradient in CLBR across EMT strata appeared to be predominantly driven by fresh-transfer outcomes, while the absolute contribution of subsequent frozen transfers was relatively stable. Future prospective studies are warranted to further validate this association and refine EMT-informed ART strategies.

## Supplementary Information

Supplementary Material 1.

## Data Availability

The data that support the findings of this study are available on request from the corresponding author, [D.M.W.], upon reasonable request.
