# Supplementary material for: Association between endometrial thickness and cumulative live birth rate in one oocyte-retrieval cycle: a retrospective cohort study including 26,127 patients
Source: BMC Pregnancy Childbirth. 2026 Mar 5;26:396. doi: 10.1186/s12884-026-08897-6 (PMC13069755; doi:10.1186/s12884-026-08897-6)
Supplement: Supplementary file 1 — Supplementary Material 1. [file 12884_2026_8897_MOESM1_ESM.docx]

**Supplemental Materials for**

Association between Endometrial Thickness and Cumulative Live Birth Rate in one oocyte-retrieval cycle: a Retrospective Cohort Study including 26127 patients

**Supplemental Figure 1.** Correlation Analysis of EMT on trigger day and EMT during FET endometrial preparation.

**Supplemental Figure 2.** CLBR and endometrial thickness by age.

**Supplemental Figure 3.** CLBR and endometrial thickness by the number of oocytes retrieved.

**Supplemental Table 1.** Clinical outcomes in fresh cycles by endometrial thickness(1mm).


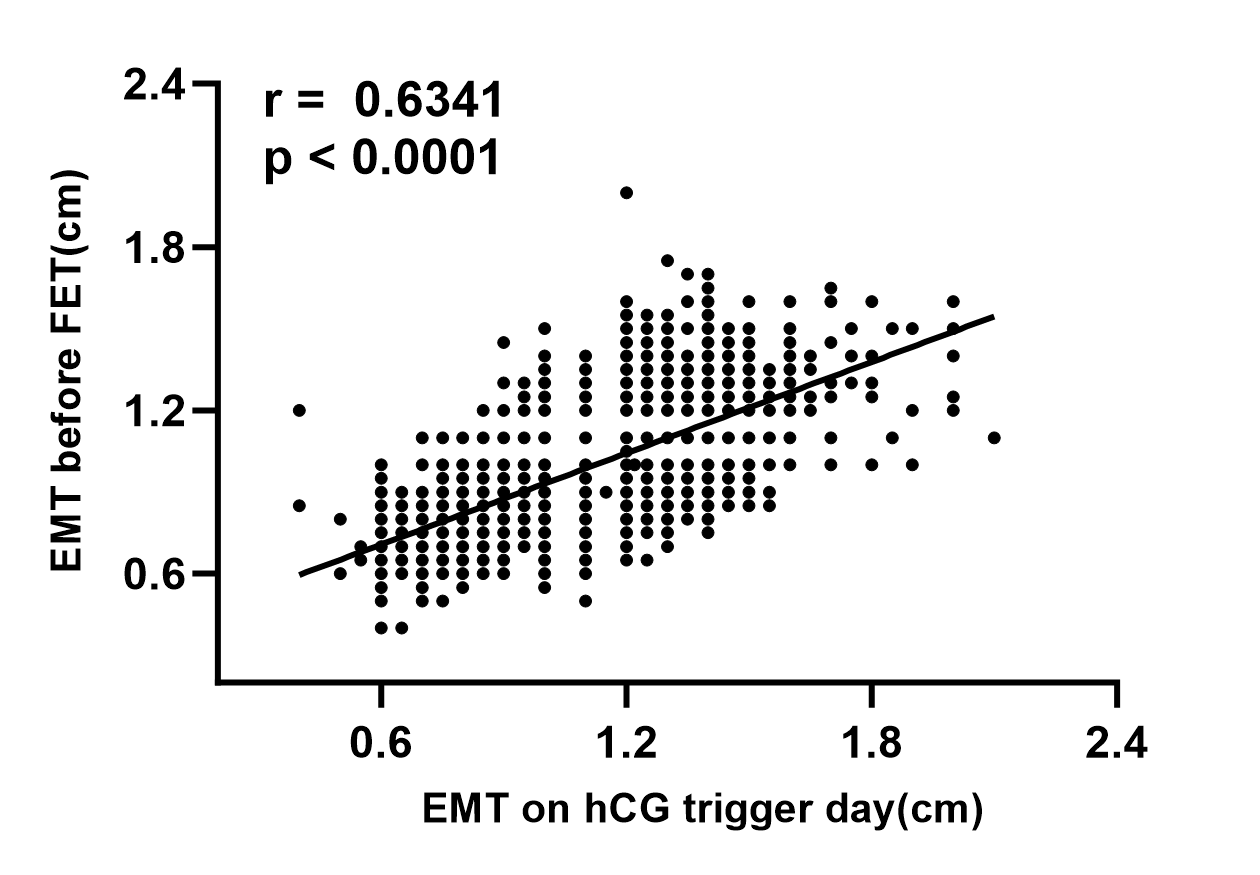


**Supplemental Figure 1.** Correlation Analysis of EMT on trigger day and EMT during FET endometrial preparation.


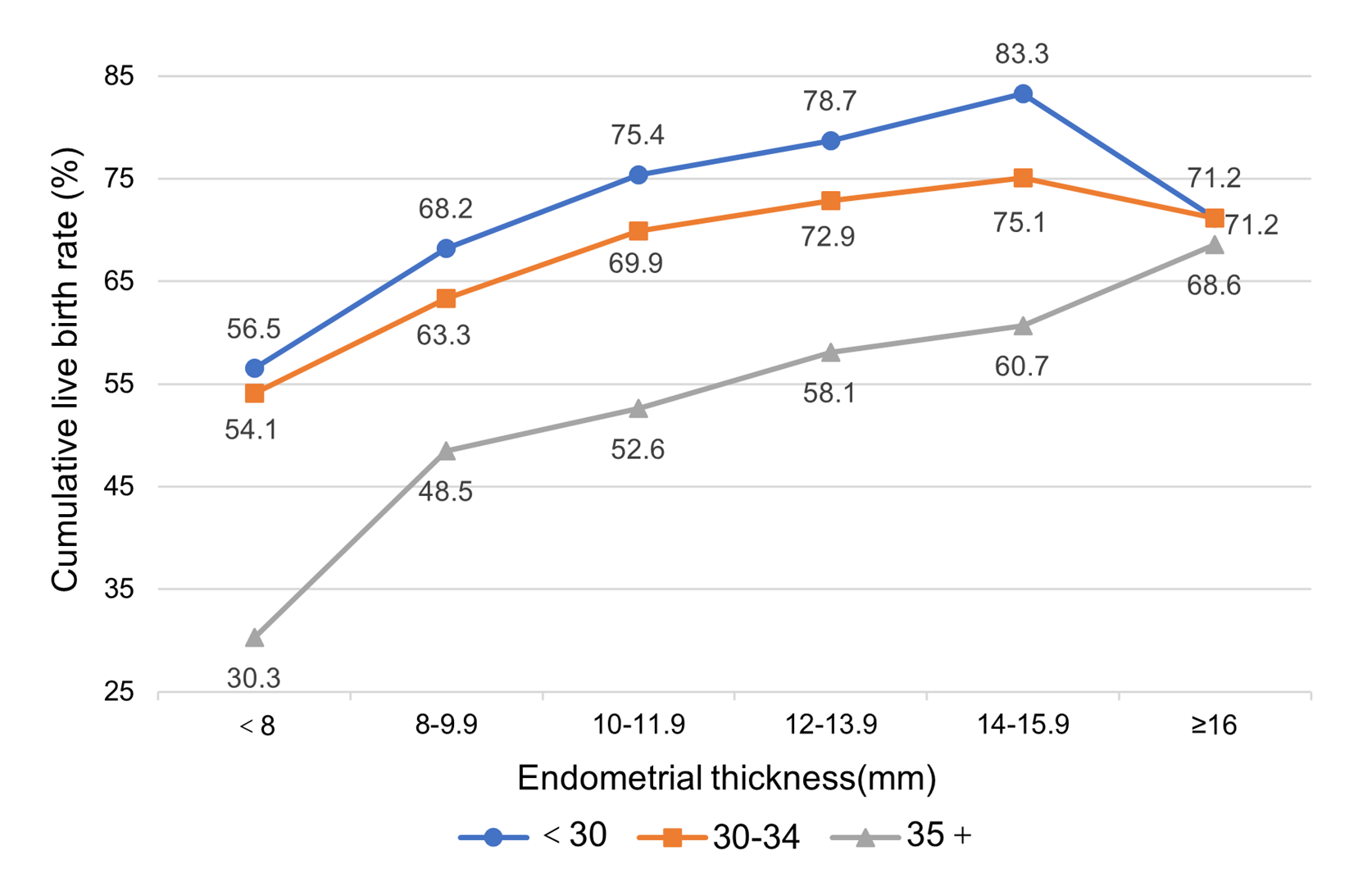


**Supplemental Figure 2.** CLBR and endometrial thickness by age.


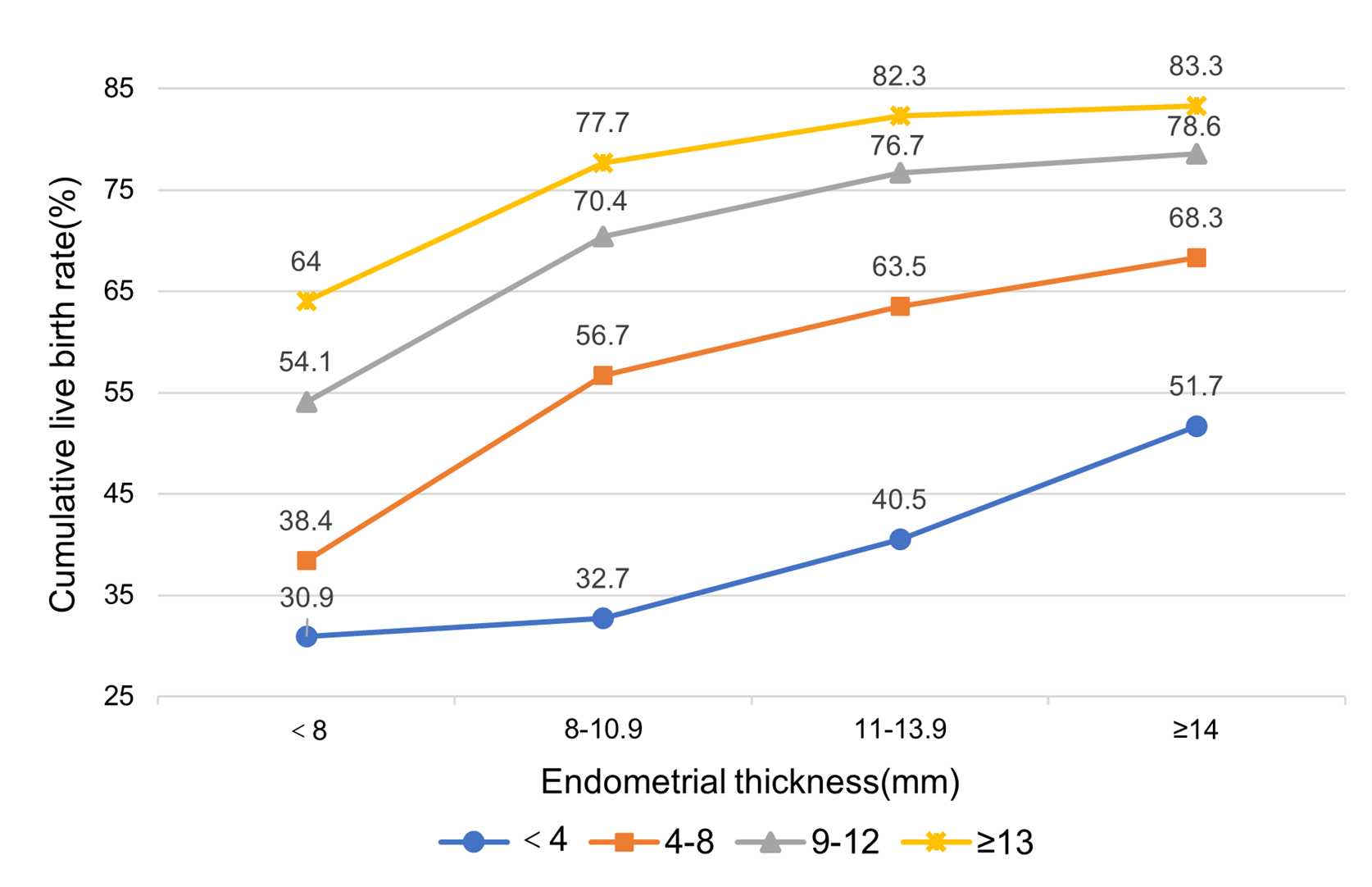


**Supplemental Figure 3.** CLBR and endometrial thickness by the number of oocytes retrieved.

**Supplemental Table 1.** Clinical outcomes in fresh cycles by endometrial thickness(1mm).

| Endometrial thickness (mm) | N | cumulative Live birth rate ,n(%) | Live birth rate ,n(%) | Biochemical pregnancy rate ,n(%) | Clinical pregnancy rate ,n(%) | Pregnancy loss rate ,n(%) |
| --- | --- | --- | --- | --- | --- | --- |
| <6 | 31 | 10(32.3) | 7(22.6) | 9(29.0) | 8(25.8) | 2(6.5) |
| 6-6.9 | 184 | 77(41.8) | 47(25.5) | 85(46.2) | 69(37.5) | 33(17.9) |
| 7-7.9 | 616 | 290(47.1) | 170(27.6) | 290(47.1) | 229(37.2) | 107(17.4) |
| 8-8.9 | 2164 | 1240(57.3) | 799(36.9) | 1162(53.7) | 989(45.7) | 321(14.8) |
| 9-9.9 | 2473 | 1557(63.0) | 1076(43.5) | 1514(61.2) | 1318(53.3) | 392(15.9) |
| 10-10.9 | 5438 | 3599(66.2) | 2555(47.0) | 3466(63.8) | 3065(56.4) | 826(15.2) |
| 11-11.9 | 4573 | 3187(69.7) | 2354(51.5) | 3065(67.0) | 2738(59.9) | 656(14.3) |
| 12-12.9 | 6051 | 4345(71.8) | 3294(54.4) | 4191(69.3) | 3791(62.7) | 846(14.0) |
| 13-13.9 | 2749 | 2018(73.4) | 1543(56.1) | 1971(71.7) | 1773(64.5) | 405(14.7) |
| 14-14.9 | 1095 | 824(75.3) | 647(59.1) | 786(71.8) | 727(66.4) | 132(12.1) |
| 15-15.9 | 447 | 339(75.8) | 255(57.0) | 316(70.7) | 288(64.4) | 58(13.0) |
| 16-16.9 | 181 | 127(70.2) | 102(56.4) | 128(70.7) | 122(67.4) | 25(13.8) |
| 17-17.9 | 55 | 37(67.3) | 30(54.5) | 39(70.9) | 36(65.5) | 8(14.5) |
| 18+ | 70 | 52(74.3) | 38(54.3) | 50(71.4) | 43(61.4) | 11(15.7) |
| total | 26127 | 17702(67.8) | 12917(49.4) | 17072(65.3) | 15196(58.2) | 3822(14.6) |
